# ASO Author Reflections: The Evolving Evidence of Prehabilitation in Frail Cancer Patients Undergoing Surgery

**DOI:** 10.1245/s10434-025-17670-6

**Published:** 2025-06-23

**Authors:** Zirong Bai, Cherry Koh, Michael Solomon, Rihan Shahab, Nicholas Hirst, Kate Alexander, Leani Souza Maximo Pereira, Ana Paula Drummond Lage, Daniel Steffens

**Affiliations:** 1 Surgical Outcomes Research Centre (SOuRCe), Royal Prince Alfred Hospital (RPAH), Camperdown, Sydney, NSW 2050 Australia; 2 Faculty of Medicine and Health, Central Clinical School, The University of Sydney, Sydney, NSW 2050 Australia; 3 Institute of Academic Surgery (IAS), Royal Prince Alfred Hospital, Sydney, NSW 2050 Australia; 4 Department of Geriatric Medicine, Royal Prince Alfred Hospital, Sydney, NSW 2050 Australia; 5 Postgraduate Program in Health Sciences, Faculdade de Ciências Médicas de Minas Gerais (FCMMG), Belo Horizonte, MG Brazil

## PAST

Frail patients undergoing cancer surgery face significant higher risks of postoperative complications, prolonged hospital stays and poor recovery.^1,2^ Despite the growing recognition of frailty as a critical predictor of adverse surgical outcomes, evidence supporting effective interventions for this vulnerable population remains somewhat limited.^3^ Recently prehabilitation interventions have been increasingly applied to this population with promising results. Therefore, there is a need to comprehensively evaluate the current literature to better determine the precise role and effectiveness of prehabilitation strategies specifically tailored for frail cancer patients facing surgical intervention.

## PRESENT

The current systematic review and meta-analysis,^4^ included randomised controlled trials exploring the effectiveness of unimodal or multimodal prehabilitation interventions consisting of physical, psychological and/or nutrition support on postoperative complications and/or length of hospital stay in patients who have been diagnosed with cancer and frailty. We found that prehabilitation significantly reduces the incidence of any postoperative complications in this high-risk population. However, its effect on other outcomes, including severity of complications and length of hospital stay, remains inconclusive due to the limited number of trials and methodological variability. These findings directly address the key clinical question by confirming that prehabilitation can improve certain postoperative outcomes in frail cancer patients, while highlighting the need for further research to clarify its broader impact.

## FUTURE

Several areas remain to be clarified in future research. Greater consistency in the use of validated frailty screening tools would improve comparability across studies and strengthen patient selection. Further investigation is also needed to determine the optimal composition, duration and mode of delivery of prehabilitation—particularly the potential role of telehealth or app-based platforms in improving accessibility and adherence. It is also important to examine whether allowing additional time for prehabilitation, by delaying surgery in selected cases, may lead to better surgical outcomes in this vulnerable group. Finally, the standardization of outcome measures in future trials may help ensure more reliable comparisons and facilitate synthesis of findings across different settings.
